# Rasch Measurement in the Social Sciences and Quality of Life Research

**DOI:** 10.5964/ejop.v11i1.913

**Published:** 2015-02-27

**Authors:** Tom Bramley

**Affiliations:** a Cambridge Assessment, Cambridge, United Kingdom

[Other p1]Rasch measurement is an approach to measuring attributes such as ‘mathematics ability’ or ‘quality of life’ – that is, the kind of attributes encountered in the non-physical sciences. It began in the 1960s in the field of educational assessment, where it is most widely known, but has spread in recent decades to other areas of social science. Within educational assessment, its main applications are in item banking, test equating and computer adaptive testing (CAT), where the focus is on estimating the level of ability/achievement/proficiency of test-takers who have taken different tests. Within social science, its main application is in developing instruments (usually questionnaires with Likert-style response formats) to measure a specific concept of research interest.

*Rasch Measurement in the Social Sciences and Quality of Life Research* is a short book for social science researchers, written with the aim of encouraging them to use the Rasch model in their own work by providing a brief introduction to the Rasch measurement perspective, discussing some of the criticisms of the use of the Rasch model, and illustrating how various kinds of research question can be approached within the Rasch framework.

The preface to the book warns readers that the aim of the book is “not to describe the models exhaustively nor to provide a step by step guide to their use”. The brevity of the book means that many of the issues it raises are not dealt with as thoroughly as they should be for it to be useful to practitioners who already know the basics of Rasch measurement. For readers without much of a quantitative background the text may well be too technical in places – terms like ‘sufficient statistics’, ‘principal components analysis’, ‘threshold estimates’ and ‘eigenvalue’ are used with little or no explanation. On the other hand, quantitative researchers familiar with statistical methods who know nothing of Rasch or IRT may want to see more discussion of how Rasch-based methods would compare and contrast with other methods they were familiar with for approaching the same kinds of research question.

Chapter 1 at 22 pages is the longest in the book, and is an introduction to the conceptual and practical issues of Rasch measurement. It draws quite heavily on other introductory textbooks. There is a good discussion of invariance and unidimensionality. Other features of Rasch models are also described, sometimes in a way which assumes a certain level of familiarity on the part of the reader. It is not emphasised clearly enough that some attractive properties of the Rasch model only apply when the data fits the model. More discussion would have been helpful of what the implications of misfit are for interpretation of results, and what the implications of addressing misfit (e.g. by removing items) are for understanding the concept being measured. There are also several pitfalls for the unwary in the form of occasional mistakes, inaccuracies and oversimplifications.

Chapter 2 is drawn from an article published by the author and colleagues in the *British Journal of Educational Research*, discussing some of the criticisms of the Rasch model and giving counter-arguments, although the brevity of the chapter means that many of the details and subtleties of the arguments for both sides cannot be explored in depth. Chapters 3 to 6 are based on other work of the author and colleagues, some of it published in this journal (i.e. *Europe’s Journal of Psychology*). They illustrate the way that different types of analysis within the Rasch framework can be used to address various problems of research interest, such as developing and refining a questionnaire, investigating unidimensionality, and investigating person misfit to the model. All the examples are interesting, but many will raise further questions in the mind of the reader which they will need to go elsewhere to answer more fully. Chapter 7 shows how the number of citations of Rasch-based articles has increased dramatically over the decades, and concludes with a summary of the basic principles and advantages of Rasch models.

Before the list of references the author gives some book suggestions, with the good advice for novice and experienced researchers to start with *Applying the Rasch Model* by Bond & Fox (2007). I would agree, and also think that enough good material is freely available online about the topics covered by the book reviewed here to make it a non-essential purchase. This book will be of most interest to those who are looking for examples and guidance relating to the analytical possibilities available within the Rasch framework when developing and validating instruments for assessing concepts of the kind found in social science research.

